# Imported loiasis in France: a retrospective analysis of 167 cases with comparison between sub-Saharan and non sub-Saharan African patients

**DOI:** 10.1186/s12879-019-4740-6

**Published:** 2020-01-20

**Authors:** Olivier Bouchaud, Sophie Matheron, Anne Loarec, Jean Dupouy Camet, Patrice Bourée, Nadine Godineau, Isabelle Poilane, Johann Cailhol, Eric Caumes

**Affiliations:** 1 Infectious Diseases and Tropical Medicine Department, Avicenne Hospital and Paris 13 University, 93000 Bobigny, France; 2 Infectious Diseases and Tropical Medicine Department, Bichat Claude Bernard Hospital and Paris 7 University, 75018 Paris, France; 3 Parasitology Department, Paris Descartes University, 75014 Paris, France; 4 Parasitology Department, Paris 11 University, 94270 Le Kremlin Bicêtre, France; 5 Parasitology Department, Saint Denis Hospital, 93200 Saint Denis, France; 6 Parasitology Department, Jean Verdier Hospital and Paris 13 University, 93140 Bondy, France; 7 Infectious Diseases and Tropical Medicine Department, Pitié Salpétrière Hospital and University Pierre et Marie Curie, 75013 Paris, France

**Keywords:** West and Central Africa, Diethylcarbamazine, Ivermectin, Loiasis, Microfilaremia, Traveller, Travel medicine

## Abstract

**Background:**

Imported loiasis is a rare cause of consultation at the return of stay in central Africa, which often poses difficult diagnostic and therapeutic questions to practitioners especially those who are unaccustomed to tropical medicine. These difficulties can lead to risks for the patients especially if inappropriate treatment is given. Large series of imported loiasis are scarce.

**Methods:**

We retrospectively studied the data including outcome in patients diagnosed with imported loiasis between 1993 and 2013 in the Paris area on the basis of a parasitological diagnosis (microfilaremia > 1/ml and/or serologic tests). We compared sub-Saharan and non sub-Saharan African patients.

**Results:**

Of the 177 identified cases, 167 could be analysed. Sex ratio was 1, mean age 41 years and 83% were sub-Saharan Africans. Cameroon was the main country of exposure (62%). Incubation time may be long (up to 18 months). Of the 167 cases, 57% presented with characteristic symptoms (Calabar swellings, creeping dermatitis, eyeworm) whereas 43% were diagnosed fortuitously. Microfilaremia was evidenced in 105 patients (63%), and specific antibodies in 53%. Compared to sub-Saharan Africans, other patients were presenting less frequently with eyeworm migration and microfilaremia whereas they had higher eosinophilia and positive serology. Prevalence of Calabar swellings was not significantly different between the two groups. Cure rates were 52% with ivermectin alone, and 77% with ivermectin followed by diethylcarbamazine. No severe adverse event was reported.

**Conclusions:**

Presentation of imported loiasis varies according to ethnicity. A systematic screening should be recommended in patients with potential exposure in endemic country. Treatment with ivermectin followed by diethylcarbamazine could be a valuable option.

## Background

Loiasis, caused by *Loa loa* and transmitted by bites of tabanid flies of the genus chrysops is endemic in the forested areas of Western and Central Africa [1–4]. Loiasis is rarely diagnosed in returning travellers being found in only 68 of 43,722 ill returning travelers (0.17%) [5]. Nine series of imported loiasis (IL) have been published over the last 30 years [6–14]. Most of them included a limited number of cases. The three largest studies including 100 cases for two of them and 186 for the third one, took place in England, Italy and the United States, respectively. In these three studies, characteristics of disease were compared between Africans and expatriates [8, 11, 13]. Diagnosis of loiasis is often difficult, and complications may be precipitated by inappropriate treatment. Indeed, in case of high microfilaremia, treatment with diethylcarbamazine (DEC) or ivermectin may lead to systemic inflammatory reactions including life-threatening encephalitis classically assigned to parasite lysis [1–3, 6, 15].

We report 167 cases observed within a 20 years-period in the Paris area with a particular attention to the differences between sub-Saharan Africans and other patients.

## Methods

We retrospectively analyzed the epidemiological, clinical, and biological data as well as treatment and outcome of all the patients diagnosed with IL between January 1993 and December 2013 in nine hospitals in Paris and its suburbs. These hospitals were selected because they are located in areas with a high density of African immigrants or they have a clinical or parasitological department involved in tropical medicine.

All the patients with a parasitological diagnosis of loiasis including positive microfilaremia (> 1/ml) and/or positive serologic tests were selected. Then, for patients diagnosed serologically, considering the limitations of serological tests, only patients with an epidemiological (stay in endemic areas) and/or a clinical presentation compatible with a loiasis were definitively included. Two populations of patients were distinguished. Sub-Saharan African (SSA) patients were defined as immigrants (born in endemic areas of sub-Saharan Africa, living in France) with a history of travel to their country of origin for visiting friends and relatives (VFR), and those living in endemic areas of sub-Saharan Africa visiting/arriving in France for various purposes. In SSA-VFR patients, we considered the last travel as that at risk of exposure to loiasis. Non sub-Saharan African (non-SSA) patients were defined as patients originating from Europe or North-Africa with a history of travel to endemic countries for loiasis. The country of acquisition was determined according to the patient’s travel characteristics. Calabar swelling was defined as recurrent and short-lasting (less than 1 week) painless oedema of the extremities (joints, legs, arms or face). Other forms of subcutaneous oedema with a different location or more prolonged duration were distinguished from Calabar swelling. Eye or subcutaneous worm migration was defined by the history of a temporary creeping lesion under the conjunctiva or the skin, leaving no trace behind, noticed by the patient and/or the physician. Ocular symptoms other than eye worm migration were analyzed separately.

Hypereosinophilia was defined by an absolute blood eosinophilic count > 500/mm^3^. Microfilaremia was determined by the microscopic observation of *Loa loa* microfilariae in a blood smear (firstly on a drop of fresh blood and secondly, when microfilariae were visualized, on a thick film after staining for confirmation and counting). In the case of negative microscopic examination, the search for microfilariae was considered negative after leucoconcentration over five milliliters was also negative. Different techniques of serology were used, each parasitology department having their own, but all considering at least two techniques for concluding to positivity including one or two screening tests and, in case of positivity, one or two confirmation techniques. Thus serology was considered positive if at least 2 tests were positive, the first being a screening and the second 1 a confirmation test. According to this rule, the different combinations of screening and confirmation techniques were as follows: i. immunofluorescence using *Molinema dessetae* antigens and/or ELISA with *Toxocara canis* antigens confirmed by an ouchterlony technique with an immuno-diffusion using *Ascaris suum* antigens and/or an immunoelectrophoresis method with *Loa loa* specific antigen; ii. direct or indirect immunofluorescence and/or ELISA confirmed by counter-electrophoresis and/or immunoelectrophoresis with *Ascaris suum* antigens; iii. Immunofluorescence using *Molinema dessetae* antigens confirmed by co-electrosyneresis using Ascaris suum antigens. Apart from the ELISA tests, which were commercial kits, these techniques were home-made and the threshold of positivity was defined by each laboratory. In case of serology classified as “undetermined” by the laboratory when it was not negative but under the threshold of positivity for each technique, we classified the result as negative.

We assessed the epidemiological data (age, sex, ethnicity, country of origin, last visited country before diagnosis, characteristics of travel), clinical aspects (medical history, reason of first consultation, description and duration of symptoms) and biological results (blood cells count, creatininemia, transaminases, filariasis serology and microfilaremia count) in patients with IL. We compared these data in SSA versus non-SSA patients, and in symptomatic versus non-symptomatic patients. We also evaluated the sensitivity of serology compared to that of direct diagnosis by microfilaremia detection for diagnosing IL.

The treatment, depending of each physician’s choice, always included ivermectin and/or DEC. DEC was given at a progressively increasing daily dosage, up to 400 mg per day, with a duration of 21 days once the dosage arrived at full dose. Full cure was defined as the absence of clinical symptoms and negative microfilaremia at the latest follow-up visit. “Partial” cure was defined as disappearance of clinical symptoms and decreased microfilaremia. Failure was stated when signs and symptoms persisted and microfilarial levels did not change significantly. Post-treatment reaction was defined by the occurrence of symptoms (fever, pruritus, angio-oedema, malaise, hypotension, fatigue, vertigo, headaches, joint or muscle or abdominal pain, central nervous system manifestations) within the 24 h following ivermectin or DEC administration.

We used Chi2 of Pearson and Mann-Whitney test (non parametric test for continuous variables) for statistical tests with Epi Info™ software (version 7.2, 2016, Atlanta, USA).

As patient data were initially collected as part of routine care and no additional examination was performed, agreement of an ethics committee was not required according to the French regulation at the time of the start of the study but the database was declared to the CNIL (Commission Nationale Informatique et Liberté). All data were completely anonymized in each of the centres that participated in the study.

## Results

Among the 177 identified cases of IL, analyzable data were available in 167, included in the present study. Sex ratio was 1.01 (84 men and 83 women), and mean age was 41.2 years (Table 1). SSA patients accounted for 83.2%. Cameroon was the leading country of exposure (62.2%), followed by Gabon and Congo-Brazzaville. Sex ratio was 0.88 among SSA patients, and 2.3 in non-SSA patients. In SSA-VFR patients and non-SSA patients, in whom the data may be calculated, the median duration of the at-risk travel was 3 months (IQR 3–30). The time between return to France and onset of symptoms could be evaluated in nine patients because they travelled in the at-risk country only once, and was estimated at 12 months (range: 6–18 months).

**Table 1:** Epidemiological and clinical characteristics in 167 patients with imported loiasis

		N	%
Age	[0–16]	9	5.3
	[16–59]	131	78.4
	[60-]	27	16.1
Sex	Female	83	49,7
	Male	84	50.2
Ethnic group	Sub-Saharan Africans	139	83,2
	Non sub-Saharan Africans^a^	28	16.7
Country of acquisition	Cameroon	104	62.2
	Gabon	27	16.1
	Congo-Brazzaville	16	9.5
	Central African Republic	6	3.5
	Others^b^	8	4.7
	Undetermined	6	3.8
Symptoms ^c,d^	Itching	74	44.3
	Calabar swelling	54	32,3
	Subcutaneous oedema	29	17.3
	Eyeworm	39	23.3
	Other ocular symptoms	24	14.3
	Subcutaneous worm migration	8	4.7
	No symptom	45	26.9

^a^Non sub-Saharan African patients = Europeans (*N* = 26) and patients from North Africa (*N* = 2)

^b^other countries: Benin, Ivory Coast, Equatorial Guinea, Mali, Rwanda, Democratic Republic of Congo

^c^total percentage of different symptoms exceeds 100% as one patient may have presented several symptoms simultaneously

^d^main data from the 3 patients who reported non-endemic countries as countries of contamination (see discussion): Ivory Coast: VFR, itching, microfilaremia: 3/mL; Mali (South): VFR, stay of 3 months, itching, microfilaremia: 4/mL; Rwanda: VFR, subcutaneous oedema (ankle), serology + with specific arc at immunoelectrophoresis

Spontaneous reporting of symptoms of loiasis motivated the initial consultation in 95 patients (56.8%). In 43.2% the diagnosis was considered because of hypereosinophilia or during systematic examination after return from endemic country evidencing suggestive symptoms (mainly pruritus) that led to diagnose loiasis by microscopy and serology. Overall 122 (73.1%) of the patients were symptomatic. Itching was present in 74 patients (44.3%). A history of creeping dermatitis was found in 8 patients. Calabar oedema were observed in 54 patients, mostly on wrists (*N* = 31) or legs (*N* = 23), and 29 patients experienced subcutaneous oedema. Eyeworm was described in 39 patients. Ocular symptoms other than sub-conjonctival crossing were pain (*N* = 6), conjunctivitis (*N* = 4), eyelid oedema (*N* = 5), ocular discomfort (*N* = 4), and other symptoms for 5 patients.

One hundred and two patients (61%) had microfilaremia with a mean value of 1822/ml (range: 1–50,000/ml) whereas 53 (31.7%) had a negative microfilaremia; data were missing in 12 cases. Ninety-two patients (55%) had a positive serology, including 54 with a specific arc evidenced by immunoelectrophoresis (performed in 125 patients). For patients for whom serology and microfilaremia results are available, 24 patients had positive microfilaremia and negative serology, 44 had negative microfilaremia and positive serology and 46 had microfilaremia and positive serology.

Clinical and biological features were compared between SSA and non-SSA patients (Table 2). Compared to SSA patients, non-SSA patients were more likely to have a prior history of loiasis (*p* = 0.01), a higher blood eosinophilia count (*p* = 0.04), and to have a positive serology (*p* = 0.04). SSA patients were more likely to have microfilaremia than non-SSA patients (*p* = 0.05), but their mean microfilaremia did not differ significantly (*p* = 0.42).

**Table 2:** Comparison between sub-Saharan African (SSA) and non sub-Saharan (non-SSA) African patients with imported loiasis

		SSA patients *N* = 139		non-SSA patients *N* = 28		p
		N	%	N	%	
Prior history of loiasis		34	24.4	14	50	0.01
Asymptomatic for loiasis		39	28.5	6	21.4	NS
Calabar swelling		43	30.9	11	39.2	NS
Eyeworm		36	25.8	3	10.7	0.05
Mean eosinophilia (/mm^3^)		1591		2854		0.04
Microfilaremia	positive	94	67.6	11	39.2	0.05
	negative	39	28	16	57.1	
	missing	7		0		
Mean^a^ microfilaremia (/ml)		2586		1247		NS
Serology	positive	64	46	24	85.7	0.04
	negative	30	21.5	2	7.1	
	undetermined	16		0		
	missing	30		1		

*NS* not significant; ^a^arithmetic means calculated on microfilaraemic subjects

Comparing biological results in asymptomatic and symptomatic patients, no difference was observed in mean eosinophilia, rates of positive microfilaremia and positive serology, and the mean number of parasites/ml (Table 3).

**Table 3:** Comparison between asymptomatic and symptomatic patients with imported loiasis

		Asymptomatic patients *N* = 45		Symptomatic patients *N* = 122		*p*
		*N*	%	*N*	%	
Mean eosinophilia (/mm^3^)		1902		2026		NS
Microfilaremia	positive	34	75.5	68	55.7	NS
	negative	9	20	44	36	
	missing	2		10		
Mean microfilaremia (/ml)		1092		2101		NS
Serology	positive	23	51.1	69	56.50	NS
	negative	9	20	17	13.9	
	undetermined	3		13		
	missing	10		23		

*NS* not significant

Diagnosis sensitivity of serology was assessed in comparison to microfilaremia detection (data not shown). Among patients with a definite diagnosis (i.e. proven by positive microfilaremia) and also with positive serology, the serology sensitivity was estimated at 69%. Sensitivity of serology among patients without microfilaremia but presenting clinical symptoms concordant with loiasis was estimated at 96%.

Outcome was evaluable in 165/167 patients including 149 treated patients and 16 patients who did not receive any treatment (loss of follow-up, pregnancy, frequent travels planned in their country of origin) (Table 4). Most patients received ivermectin alone (75.8%) or followed by DEC (17.4%) whereas 10 patients (6.7%) received DEC only. Ivermectin was given either as a single course (7.1%) or repeated courses (92.9%). A preventive treatment of post-treatment reaction (anti-histaminic and/or corticosteroids) was given in 26 patients. Mean time of follow-up was 6 months (range: 1–34 months). Full cure rate was 52.2% in the patients treated with ivermectin alone (1 to 6 courses), and 76.9% in those who received ivermectin followed by one course of DEC. Eleven patients (7.3%) experienced post-treatment reaction (4.4% following ivermectin alone, 20% following DEC alone, 15.3% following ivermectin + DEC), consisting in fever, malaise, fatigue, headache, and/or abdominal pain. No severe adverse event (including encephalopathy) was reported. No post-treatment reactions were reported in patients who received preventive treatment with anti-histaminic and/or corticosteroids.

**Table 4:** Treatment outcomes in 149 patients with imported loiasis

	ivermectin^ab^		diethylcarbamazine^cd^		ivermectin^a^ then diethylcarbamazine^c^	
	*N* = 113 (75.8%)		*N* = 10 (6.7%)		*N* = 26 (17.4%)	
	*N*	%	*N*	%	*N*	%
Outcomes:						
failure	10	8.8	2	20	2	7.6
full cure	59	52.2	0	0	20	76.9
partial cure	0	0	5	50	2	7.6
loss of follow up	44	38.9	3	30	2	7.6
preventive treatment of post-treatment reaction	15	13.2	4	40	7	26.9
post-treatment reaction	5	4.4	2	20	4	15.3

^a^between 1and 6 courses (1 course: 62.8%; 2: 17.8%; 3: 7.7%; 4: 6.2%; 5: 2.3%; 6: 3.1%), 10 (7.1%) patients received only 1 course, associated with albendazole in 4 cases

^b^200 μg/kg per course

^c^progressive dosage (initial dosage between 10 and 75 mg, final dosage 200–400 mg for 21 days)

^d^Two patients with a high microfilaremia (38,200 and 50,000/mL, respectively) were treated with filariopheresis followed by diethylcarbamazine without significant adverse event

## Discussion

This large study of imported loiasis shows that loiasis may be asymptomatic, may appear long time after return (up to 18 months) and that the hypothesis of persistent low transmission in formerly endemic areas could be further investigated. It also contributes to assess the response to treatment although the limitations of a retrospective study should lead to caution in interpreting these results.

In our study the leading country of acquisition of IL was Cameroon, in agreement with the results of two other French studies [6, 10]. In England, the leading country of acquisition is Nigeria [8]. This is not surprising as loiasis is highly endemic in Cameroon and Nigeria, and as both countries account for a high number of migrants in France, and England respectively, according to colonial history.

However four patients were found to have been infected in countries where loiasis is not currently considered to be endemic (Mali, Benin, Ivory Coast, Rwanda) even if a limited focus of loiasis has been described in the south-east part of Benin [1, 2, 16]. This has not been showed in other studies of IL. According to some authors, the western part of the African rain forest has been considered in the past as a possible endemic zone for loiasis [2, 16]. Although it is most likely that these patients have forgotten to mention a stay in an endemic area, it is possible to hypothesize persistent transmission at a low level in isolated areas of these formerly endemic areas.

Studies of IL usually fail to determine incubation period since it is not possible to estimate the date of acquisition neither in patients native from endemic country nor in long term travellers or travellers traveling frequently to endemic areas. We estimated the median incubation time at 12 months in the nine patients with data allowing this evaluation. If this median incubation time cannot be extrapolated to all patients in the study, it is important to note for the clinician that clinical signs of loiasis may appear long after return and up to 18 months in our study. This duration is in agreement with that found in the only other study in which this parameter was evaluable [6].

A high proportion (43.2%) of our patients was fortuitously diagnosed because history of compatible but mild or non specific symptoms and/or hyperereosinophilia after returning from endemic countries. This point has not been highlighted in other series of IL because patients were generally included only on the basis of specific symptoms [1–3]. As a result we recommend that every patient at risk of loiasis (ie: having lived or travelled for a long time in endemic areas even long time ago) should be systematically screened for loiasis.

Overall 73% of our patients were symptomatic, with classic symptoms (itching, Calabar oedema, creeping dermatitis, eye worm) but also with less characteristic clinical manifestations. Itching was a complaint in nearly two thirds of our symptomatic population, compared to one third of that reported by Churchill et al. [8] Calabar oedema and migratory oedema were reported in respectively 44 and 24% of our symptomatic patients, whereas Calabar swellings were reported in, respectively, 62 and 74.7% of symptomatic cases by Churchill and Herrick [8, 13]. However, we differentiated sensu stricto Calabar oedema from migratory oedemas which can explain such difference [17]. We also differentiated ocular symptoms from eye worm migration because one of our patients presented an intra-vitreous haemorrhage, a complication rarely reported [3, 18, 19]. Creeping dermatitis was found in about 5% of our patients but loiasis is a very rare cause of creeping dermatitis, been found in only one amongst 70 patients consulting for creeping dermatitis [20].

We found some significant differences related to ethnicity as non-SSA patients were found with less frequent eyeworm, higher eosinophilia, fewer detectable microfilaremia, and more common positive serology compared to SSA patients. We thus confirm the results found in five other comparative studies [8, 10–13]. Such differences have been previously described and were attributed to a possible immune tolerance in Africans with multiple exposures to the parasite [2, 7, 8, 10–13, 21–24]. Some authors highlighted the major role played by the antibodies-mediated immune response (with notably specific IgG antibodies) in cooperation with cellular immunity including lymphocyte proliferation to parasites antigens [7, 12, 16, 25]. In keeping with this hypothesis, we found that the sensitivity of serology was higher among patients without detectable microfilaremia, suggesting an immune mechanism which controls the multiplication of parasites. Similar results were found by Churchill with a better sensitivity of serology in expatriates compared to Africans [8]. Herrick hypothesises that differing eosinophil-associated responses to the parasite may be responsible for the differences in clinical presentations [13].

Current diagnostic tools have limitations and more effective tests are needed in both endemic areas or in the frame of IL. Recently, a rapid antibody-detection test has been developed [26]. Should the first encouraging results be confirmed, this new tool would certainly be most useful to diagnose loiasis, especially in its occult (amicrofilaraemic) forms.

One or more courses of ivermectin, alone and followed by one course of DEC, gave a cure rate of 52 and 77%, respectively, with a low rate of adverse events and no severe adverse event. Similar results have been found in smaller studies. Churchill et al. showed a cure rate of 63% (with no difference between Africans and non-Africans) and a relapse rate of 12% among 100 patients who were mainly treated with diethylcarbamazine [8]. Klion et al. reported, in 32 expatriates followed up during a median time of 4.5 years, a cure rate of 38% after one course of DEC, and 16% after two courses, whereas 53% relapsed, within the first year for the majority [7]. El Aouri reported eight relapses (31%) among 26 expatriates returning from Equatorial Guinea and treated with DEC (*N* = 15), ivermectin plus DEC (*N* = 9) or ivermectin alone (*N* = 2) [9].

DEC is the corner-stone of the treatment of loiasis due to its macrofilaricidial activity (in contrast to ivermectin or albendazole). Therefore, in patients living in non-endemic countries, at least one course of ivermectin followed by one course of DEC appears to be a good option to reach an acceptable cure rate without taking the risk of severe adverse event. This is consistent with a 93% reduction of microfilaremia observed in seven patients treated by ivermectin before receiving DEC. [27] This option seems particularly adequate when microfilarial density is relatively high (between 2000 and 8000/ml) while a density below 2000/ml allows to initiate the cure directly with DEC according to Boussinesq [28]. Adverse events following DEC (and at a lesser extent ivermectin) have been reported both in endemic zones and in IL [1–3, 6, 8, 15]. The higher is the parasite load the higher is the risk of developing marked or serious adverse events such as encephalopathy when microfilaremia is above 50,000/ml [9, 12]. Recent data suggest that post-treatment reactions following DEC and ivermectin occur earlier with DEC but share a common pathophysiology [29].

Our study has some limitations that mostly concern inclusion criteria and treatment mainly due to the retrospective design of the study. The first limitation is the lack of standardization of diagnostic tests because, if the criterion of positivity was the same for all patients (at least 2 positive serological tests including a screening test and a confirmation test), the techniques used and the positivity thresholds varied from one centre to another. However, since for patients diagnosed by serology alone we only considered those with an epidemiological and clinical history compatible with a loiasis diagnosis and excluded cases with serologies for which the result was not clearly positive, we believe that the risk of mis-diagnosis is limited. Another limitation is the heterogeneity of treatment regimens, the number of patients lost to follow up, and the limited duration of treatment follow up, but these limitations are shared by other studies that had fewer patients than ours.

## Conclusions

We recommend to systematically consider loiasis in all patients returning from endemic countries with either hypereosinophilia, pruritus or recurrent oedema in addition to the more classic signs, even several months or years after return. The association of one or more courses of ivermectin followed by at least one course of DEC appears a valuable option for treating imported loiasis and needs to be evaluated as well as the use of albendazole which has not been assessed in this setting.

## Data Availability

The datasets used and/or analysed during the current study are available from the corresponding author on reasonable request.

## Abbreviations

DEC: Diethylcarbamazine
IL: Imported loiasis
IQR: Interquartile
SSA: Sub-Saharan African patients
VFR: Visiting friends and relatives
